# Midline 1 associated with Fas signaling enhances murine antigen-induced arthritis

**DOI:** 10.3389/fcell.2025.1451093

**Published:** 2025-05-15

**Authors:** Nina Lukač, Darja Flegar, Sara Priselac, Tomislav Kelava, Alan Šućur, Maša Filipović, Dino Šisl, Martina Fadljević, Igor Radanović, Vedran Katavić, Nives Zimmermann, Danka Grčević, Nataša Kovačić

**Affiliations:** ^1^ Laboratory for Molecular Immunology, Croatian Institute for Brain Research, University of Zagreb School of Medicine, Zagreb, Croatia; ^2^ Department of Anatomy, University of Zagreb School of Medicine, Zagreb, Croatia; ^3^ Department of Physiology and Immunology, University of Zagreb School of Medicine, Zagreb, Croatia; ^4^ Division of Allergy and Immunology, Cincinnati Children’s Hospital, and University of Cincinnati College of Medicine, Cincinnati, OH, United States

**Keywords:** rheumatoid arthritis, myeloid lineage, midline 1, Fas, inflammation

## Abstract

**Introduction:**

Rheumatoid arthritis is the most common immune-mediated joint disease, whose pathogenesis includes both innate and acquired immune mechanisms. Fas/Fas ligand system is considered to have a dual role in arthritis, inducing apoptotic cell death of hyperplastic synoviocytes and inflammatory cells, but also exerts proinflammatory effects. In our study, absence of Fas resulted in decreased accumulation of myeloid cells in affected joints.

**Methods:**

Proportions of synovial hematopoietic cells were assessed by flow cytometry in wild-type and *Fas −/−* mice with antigen-induced arthritis. Effects of myeloid-specific ablation of Fas on the course of antigen-induced arthritis was assessed using Fas^fl^/LysMCre model. Arthritis was scored visually, histologically and by micro-computerized tomography. Transcriptome of sorted CD11b^+^Gr-1^+^ cells was analyzed by microarray, and effects of potential molecular driver Midline-1 (Mid-1) were analyzed *in vitro* and using *Mid1 −/−* mice.

**Results:**

Ameliorated antigen-induced arthritis in *Fas −/−* mice is characterized by the lack of synovial accumulation of myeloid CD11b^+^Gr-1^+^ cells. However, myeloid-specific ablation of Fas was not sufficient to ameliorate arthritis, suggesting proinflammatory effects of Fas in multiple cell subsets in arthritis. Myeloid cells from *Fas −/−* mice downregulated limited number of genes including *Mid1*. Stimulation of bone marrow cells with low doses of soluble Fas agonist upregulated expression of *Mid1*. Inactivation of *Mid1* had a variable anti-inflammatory effects *in vitro* and partial anti-arthritic effect *in vivo*.

**Conclusion:**

Functional Fas is required for the recruitment and accumulation of innate inflammatory cells in arthritic joints. This accumulation is not driven exclusively by mediators expressed in accumulated subset. *Mid1* enhances inflammatory polarization of myeloid cells and promotes bone and cartilage degradation in arthritis.

## 1 Introduction

Rheumatoid arthritis is the most common form of immune-mediated joint destruction, whose manifestation depends on the complex interplay of various cells and mediators (McInnes and Schett, 2007). The disease mechanisms largely depend on the activation of lymphocytes responsible for the recognition of auto-antigens and production of auto-antibodies (Fournier, 2005), but also on the activation of innate inflammatory cells that act as an important contributor to the initiation and progression of many autoimmune diseases (Morell et al., 2017; Takaki-Kuwahara et al., 2019). One of the suggested anti-inflammatory mechanisms is apoptosis of inflammatory cells mediated by Fas (CD95, Apo-1), a typical death receptor and a regulator of immune system homeostasis through the phenomenon of activation induced cell death (French and Tschopp, 2003; Askenasy et al., 2005). However, Fas exerts a dual role in arthritis. It has the ability to hamper the synovial hyperplasia by inducing apoptosis of hyperplastic synoviocytes and inflammatory cells, but also to promote the disease (Calmon-Hamaty et al., 2015). Ligation by soluble Fas ligand, cleaved from the membrane by metalloproteinases (O' Reilly et al., 2009) may activate non-apoptotic signaling pathways, such as nuclear factor κB (NF-κB) or Src kinase c-yes (Xiao et al., 2002; Buchbinder et al., 2018). In hyperplastic synoviocytes, soluble Fas ligand is able to activate ERK1/2, PI3K, caspase 8 and JNK pathways and induce their proliferation, while blockage of JNK pathway reverts the response to soluble Fas ligand to apoptosis (Audo et al., 2014). Soluble Fas ligand is also able to stimulate integrin-dependent adhesion of CD11b^+^Gr-1^+^ neutrophils to vascular endothelium via Syk-Btk/PLCγ2/Rap1 signaling pathway in autoimmune conditions (Gao et al., 2016). Several *in vivo* studies confirmed the proinflammatory role of Fas in arthritis. DBA1/*lpr* mice, with spontaneous mutation of Fas are resistant to collagen-induced arthritis, which has been ascribed to inefficient stimulation of hyperplastic synoviocytes (Tu-Rapp et al., 2004). A later study by the same authors described inefficient activation of macrophages by the IL-1R or TLR4 (Toll-like receptor 4) in conditions of blocked Fas signaling, due to interaction of FADD with the TLR adaptor protein MyD88, and suggested that Fas ligation promotes activation of the IL-1R-TLR pathways (Ma et al., 2004). Our group has also described ameliorated non-destructive antigen-induced arthritis (AIA) in mice with a gene knockout for Fas (*Fas −/−*, Adachi et al., 1995) with preserved bone and cartilage architecture, potentially due to decreased apoptosis of osteochondroprogenitor populations (Lazić Mosler et al., 2019). These mice also showed suppressed joint inflammation. To unravel the potential mediators of Fas proinflammatory and proresorptive signaling, in this study we characterized frequencies of main hematopoietic articular populations in Fas-deficient mice with AIA, analyzed the transcriptome of CD11b^+^Gr-1^+^ cells as the most altered subset, and assessed the role of downregulated midline 1 (*Mid1*) gene as a potential proinflammatory and proresorptive mediator in arthritis.

## 2 Materials and methods

### 2.1 Mice

Eight to 18-week old female C57BL/6J wild-type (wt) mice, mice deficient in the Fas gene (*Fas −/−*) and mice deficient for Midline 1 (*Mid1 −/−*) and 8-12-week old male and female mice with conditional ablation of Fas in myeloid lineage (Fas^fl/fl^x LysMCre^+/−^) all on the C57BL/6 background were used in experiments. The *Fas −/−* mice (Adachi et al., 1995) and wt control (C57BL/6) strain were a gift from Dr. M. Simon (Max Planck Institute for Immunobiology, Freiburg, Germany). The Fas^fl/fl^ mice (Stranges et al., 2007) were a gift from Dr. D. Konrad, Division of Pediatric Endocrinology and Diabetology, University Children’s Hospital, Zurich Center for Integrative Human Physiology, University of Zurich, Switzerland. The *Mid1 −/−* mice and wt control (C57BL/6) strain were a gift from Dr. G. Meroni (Department of Life Sciences, University of Trieste, Trieste, Italy). LysMCre mice (The Jackson Laboratory, stock no. 004781) that express Cre recombinase in myeloid lineage cells, were crossed with Fas^fl/fl^ mice to generate Fas^fl/fl^LysM-Cre^+/−^ mice which were then crossed with Fas^fl/fl^ strain to obtain mice with the conditional ablation of Fas in myeloid lineage (Fas^fl/fl^LysM-Cre^+/−^) and controls (Fas^fl/fl^LysM-Cre^−/−^). All colonies were bred and maintained at the animal facility of the Croatian Institute for Brain Research, University of Zagreb, School of Medicine, under standard conditions (10 h light and 14 h dark daily, standard chow (4RR21/25; Mucedola, Italy) and water *ad libitum*). All animal protocols were approved by the Ethics Committee of the University of Zagreb, School of Medicine (380-59-10106-15-168/235, 380-59-10106-19-111/250) and the National ethics committee (EP 07-2/2015, EP 230/2020) and conducted in accordance with accepted standards of ethical care and use of laboratory animals.

### 2.2 Antigen-induced arthritis (AIA)

**FIGURE 1:**
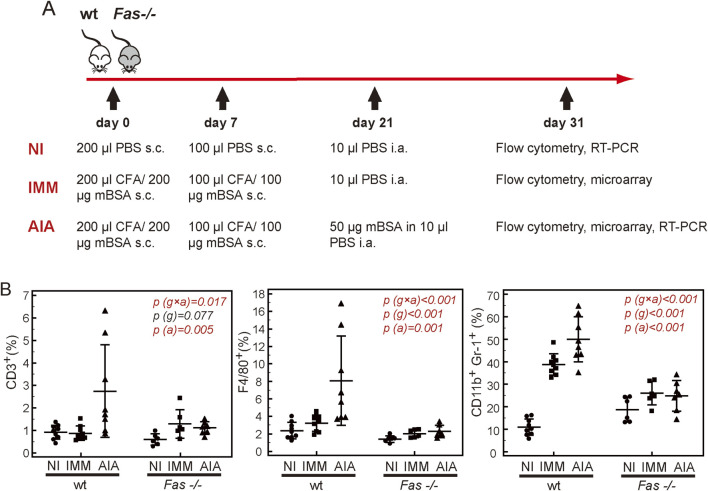
Synovial hematopoietic populations in wild-type (wt) and Fas-deficient mice (*Fas −/−*). **(A)** Schematics of experimental design: Initial immunophenotyping was performed in individual non-immunized (NI), immunized control (IMM) and mice with arthritis (AIA) (n > 6). Experiments were repeated 3 times. The data on the frequencies of cell populations in relation to µCT parameters, were collected as a part of a wider overarching study characterizing the course of arthritis in Fas-deficient mice (Lazić Mosler et al., 2019). Microarray analysis included individual samples of sorted CD11b^+^Gr-1^+^cells from four IMM and AIA wt and *Fas −/−* mice, aiming to assess their profile during general immune activation vs. antigen-specific immune-mediated arthritis. RT-PCR validation included RNA samples from bulk-sorted pooled CD11b^+^Gr-1^+^cells from minimum 4 NI and 4 AIA wt and *Fas −/−* mice, or knee tissue extracts from individual NI and AIA wt and *Fas −/−* mice (n ≥ 4) to confirm alterations of genes of interest during general immune activation. **(B)** Mice were sacrificed on day 10 following intra-articular (i.a.) injection, and single cell suspensions were labelled with anti-mouse Gr-1-PE, anti-CD11b-PECy7, anti-CD3-APC, and anti-F4/80- APCeF780, and 7-amino-actinomycin D (7-AAD). Proportions of T lymphocytes (CD3^+^), macrophages (F4/80^+^), and granulocytes-monocytes (CD11b^+^, Gr-1^+^), were determined amongst live cells. Horizontal lines and bars, mean ± SD; p, two-way ANOVA, g, genotype factor, a, arthritis and immunization factor.

AIA was chosen for its clearly defined onset, localization, and high incidence, ensuring reliable group comparisons and precise sample timing and its suitability for C57BL/6 strain used in this study. Presence of periarticular bone destruction further makes it ideal for investigating its pathogenesis. Because it affects the knee joints rather than just small joints it allows for precise characterization of subchondral bone and cartilage damage. AIA was induced as described previously (Lazić Mosler et al., 2019) (Figure 1A). Briefly, mice were immunized by two subcutaneous (s.c) injections of methylated bovine serum albumin (mBSA, #A1009, Sigma-Aldrich, St Louis, MO, United States) emulsified in complete Freund’s adjuvant (CFA, Sigma-Aldrich). Mice were randomly assigned to one of the experimental groups. Non-immunized control mice (NI) were injected s.c. with 0.1M phosphate buffered saline (PBS). Arthritis was induced 3 weeks after the initial immunization by intra-articular (i.a.) injection of mBSA dissolved in PBS into both knees, under tribromoethanol (Sigma-Aldrich) or ketamine (Richter Pharma AG, Wels, Austria)/xylazine (Alfasan International BV, Woerden, Netherlands) induced anesthesia. Immunized (IMM) and NI controls were injected i.a. with 10 μl of sterile PBS. Mice were sacrificed on day 31 post-immunization (day 10 post-i.a. injection) by cervical dislocation under tribromoethanol or ketamine/xylazine anesthesia, and transverse diameters of each knee were measured 3 times by a vernier scale/caliper. Knees were then harvested for flow cytometry (performed in all NI, IMM, and AIA mice), FACS sorting for microarray analysis (IMM and AIA mice), and validation of microarray results (NI and AIA mice, Figure 1A). The data on the frequencies of cell populations in relation to micro-computerized tomography (µCT) parameters were collected as a part of a wider overarching study characterizing the course of arthritis, inflammation, bone features, and overall synovial cellular composition of Fas deficient mice with arthritis (Lazić Mosler et al., 2019). Arthritis was assessed by 4-point visual score (0 - no arthritis, 1 - discrete localized thickening of the joint capsule, 2 - mild swelling, absence of sharp patellar ligament contour, 3 - clear swelling with diffuse thickening of the joint capsule, 4 - severe swelling and deformity, visible through the skin) and by measuring knee diameters. Knees were then harvested for μCT, RT-PCR, and flow cytometry.

### 2.3 Histology and RNAScope

Knee joints were dissected, released from soft tissues, fixed in 4% formaldehyde at 4°C for 24 h and decalcified for 2 weeks in 14% ethylene-diamine tetra-acetic acid and 4% formalin, then dehydrated, and embedded in paraffin. Frontal 6 μm sections through the knee joint were cut with a Leica SM 2000 R rotational microtome (Leica, Nussloch, Germany). Histological assessment of arthritis severity was performed on sections stained with Goldner-trichrome or toluidine blue, by semi-quantitative scoring described previously (Lazić Mosler et al., 2019). *In situ* hybridization was performed using RNAscope® kit (Wang et al., 2012) in ACD HybEZTM II Hybridization System (220V) and ACD EZ-Batch Slide System (Advanced Cell Diagnostics, Inc, Minneapolis, MN, SAD), according to the manufacturer’s instructions*. Mid1* expression was detected using a specific probe (RNAscope® Probe - Mm-Mid1). RNAscope® Positive Control Probe (Mm-Polr2a) and RNAscope® Negative Control Probe (DapB) were applied to same level sections. Probe binding was detected by horseradish peroxidase substrate diaminobenzidine (DAB). Brown DAB precipitate and hematoxyline counterstained nuclei were visualized using Axio Imager microscope (Carl Zeiss Microscopy GmbH, Jena, Germany).

### 2.4 Micro-computerized tomography

The distal femora were scanned using a μCT system SkyScan 1076 (Bruker, Kontich, Belgium), at 50kV, 200 μA, and a 0.5 mm aluminum filter, detection pixel size 9 μm, and image capture every 0.4° (for *ex vivo* scans) or every 1.2° (for *in vivo* scans) through 180° rotation of the camera. The scanned images were reconstructed using Recon software (Bruker) and analyzed using CTAn software (Bruker). Three-dimensional analysis and reconstruction was performed as described previously (Lazić Mosler et al., 2019), assessing trabecular bone volume (BV/TV, %), trabecular number (Tb.N, mm^-2^), trabecular thickness (Tb.Th, µm), and trabecular separation (Tb.Sp, mm).

### 2.5 Flow cytometry and cell sorting

Isolated joints (excision was performed at the level of growth plates) were cleaned from the surrounding muscles and injected with 1 mg/ml collagenase type IV (Sigma-Aldrich) and incubated for 1 h at 37°C. Single cell suspensions were prepared by passing the cells through a 100 μm-cell strainer into FACS tubes. Non-specific antibody binding was blocked by anti-mouse CD16/CD32 (BioLegend, RRID:AB_1574975) for 5 min at RT, and after that cells were labeled by the addition of following antibodies: anti-CD45RA(B220)-FITC (BioLegend, RRID:AB_312991), anti-Ly-6G/Ly-6C(Gr-1)-PE (eBioscience, RRID:AB_466045), anti-CD11b-PECy7 (BioLegend, RRID:AB_312799), anti-CD3-APC (BioLegend, RRID:AB_312677), and anti-F4/80-APCeFluor780 (eBioscience, RRID:AB_2735036), anti-CD95-AF488 (eBioscience, RRID:AB_10671269) and/or anti-CD95-FITC (BD Biosciences, RRID:AB_395329), or corresponding isotype controls (mouse IgG1κ-AF488, eBiosciences, RRID:AB_470230, Armenian hamster IgG2λ-FITC, BD Biosciences, RRID:AB_395165). Dead cells were excluded by binding of 7-amino-actinomycin D (7-AAD, BioLegend) or 4′,6-diamidino-2-phenylindole dihydrochloride (DAPI, Sigma-Aldrich). Proportion of apoptotic cells was determined by binding of Annexin V (BioLegend) and 7-AAD staining, according to the manufacturer’s instructions. Scatter and fluorescent signals were acquired on Attune (Thermo Fisher Scientific) instrument and analyzed by FlowJo software (FlowJo v10, Ashland, OR, United States). Cell sorting was performed by a BD FACSAria I and IIu (BD Biosciences, Franklin Lakes, New Jersey, United States) instrument, as described previously (Ikić Matijašević et al., 2016). CD11b^+^Gr-1^+^ cells were sorted in 2 ml collection tubes containing PBS with 2% FCS and used for RNA extraction. Sorting parameters were optimized for high-purity sorting. Sorting purity was determined by a re-analysis of sorted population and was greater than 99.5% for all experiments.

### 2.6 Cell culture and treatment

Bone marrow cells isolated from wt or *Mid1 −/−* mice were plated in 48- or 96-well culture plates at a density of 1–2 × 10^6^ cells/0.2–0.5 ml per well of α-MEM supplemented with 10% FCS, (Gibco, Thermo Fisher Scientific). Cell activation was induced by overnight incubation with 10 µg/mL of heat-inactivated *Mycobacterium tuberculosis* (Difco) at 37°C. In a separate set of experiments cells were, prior to activation, treated with 20–100 µM GSK′364A (Cambridge Research Biochemicals, Billingham, UK), a peptide antagonist of Mid1-PP2A interaction for 1h/37°C. For the assessment of apoptotic and non-apoptotic effects of Fas, cells were treated with 0.01–1 μg/mL anti-mouse CD95 antibody (BD Biosciences, RRID:AB 395326) or the corresponding isotype control (Armenian hamster IgG2λ, BD Biosciences RRID:AB_395162) with or without the addition of 1 μg/mL protein G (Sigma-Aldrich) and incubated for 16–18 h. Untreated cells or cells treated only with 1 μg/mL protein G were used as controls.

### 2.7 RNA isolation and gene expression analysis

RNA was extracted by Trizol (Thermo Fisher Scientific), as described previously (Kovačić et al., 2007). For microarray analysis samples were pre-cleaned by MagMAX™-96 for Microarrays Kit (Ambion, Thermo Fisher Scientific), and quality assessed by Agilent total RNA Pico chip (Agilent technologies). RNA was then amplified by GeneChip™ WT Pico Kit (Affymetrix, Thermo Fisher Scientific), and cRNA was transcribed, fragmented, labeled and individual samples hybridized to Mouse ST 2.0 arrays (Affymetrix).

### 2.8 Real-time polymerase chain reaction

Total RNA was converted to cDNA by reverse transcriptase (Thermo Fisher Scientific), and amplified by an ABI 7500 instrument (Applied Biosystems, Thermo Fisher Scientific), using commercially available TaqMan Assays (mouse *β-actin*, Mm00607939_s1; *Erdr1* Mm04214945_uH; *Fas*, Mm00487425_m1; *IL-1β*, Mm00434228_m1; *IL-6*, Mm00446190_m1; *Mid*1, Mm01166432_m1 *Thbs1* Mm00449018_m1; *TNF*, Mm00443258_m1), as described previously (Kovačić et al., 2007). Samples were amplified individually, while pooled cDNA samples used for construction of standard curve for relative quantification were amplified in duplicates. Negative controls containing RNAse free water instead of sample were used to exclude potential contamination during reverse transcription and PCR amplification.

### 2.9 Western blot analysis

For protein extraction, knees were homogenized in 1 ml of cell lysing buffer (Cell Signaling Technologies) supplemented with 1 µl Halt Protease Inhibitor Cocktail (Thermo Fisher) and 1 µl Halt Phosphatase Inhibitor Cocktail (Thermo Fisher), lysed by sonication, centrifuged at 14,000 g/10 min/4°C. Supernatants were mixed with NuPAGE® LDS sample buffer (Thermo Fisher) and NuPAGE® reducing agent (Thermo Fisher), denatured at 95°C for 5 min, and proteins separated on 12% stain free gels prepared with TGX Stain-Free^TM^ FastCast Acrylamide Kit (Bio-Rad) by PAGE-SDS electrophoresis. After gel activation (5 min/UV), proteins were transferred onto a methanol activated PVDF membrane. Total protein amount on membrane was assessed by membrane imaging after brief UV excitation. After blocking with 5% dry milk in 0.1% PBST for 1h/RT, membranes were incubated with rabbit anti-Mid1 antibody (1:500, Thermo Fisher Scientific Cat# PA5-36305, RRID:AB_2553463), overnight at 4°C, and then with HRP conjugated anti-rabbit antibody (1:50,000, Jackson ImmunoResearch) for 1h/RT. Antibody binding was visualized with SuperSignal™ West Femto Maximum Sensitivity Substrate (Thermo Fisher) using ChemiDoc MP™ imaging system (Bio-Rad). Signal intensity was quantified and normalized to total protein signal intensity, using stain free technology (Rivero-Gutiérrez et al., 2014), in Image Lab Software (Bio-Rad).

### 2.10 Data analysis and interpretation

Data are plotted as individual values, horizontal lines and markers represent means ± SD or medians (IQR). Statistical analysis was performed by MedCalc Statistical Software version 12.5.0 (MedCalc Software bvba, Ostend, Belgium). Depending on the number of factors, distribution, variable type, and the number of comparisons, data were analyzed by t-test and one or two-way ANOVA, or Mann-Whitney and Kruskal-Wallis test. Kolmogorov–Smirnov test was used to assess the normality of the data.

Microarray analysis was done with R, using Bioconductor software (https://bioconductor.org). Values were pre-processed by robust multi-array average (RMA) algorithm. Quality of chips was assessed before and after normalization, showing similar intensities for all chips, and minor variations corrected by normalization. Relative log expression (RLE) was grouped at 0, whereas normalized unscaled standard error (NUSE) was grouped around 1. Correlation coefficients of individual chips were 0.93 and 0.96, before and after processing, respectively. Principal component analysis (PCA) and hierarchical clustering excluded the batch effects. Differences in gene expression were assessed by linear models for microarray analysis (*limma*) package (Ritchie et al., 2015). Microarray data are available on the GEO repository (Gene expression of sorted synovial myeloid cells from wild type and Fas −/− mice with antigen-induced arthritis and immunized control mice without arthritis, accession number GSE141592) (Kovačić and Lukač, 2019).

## 3 Results

### 3.1 Ameliorated AIA in *Fas −/−* mice is characterized by the lack of accumulation of myeloid CD11b^+^Gr-1^+^ cells in synovial compartment

We have previously described reduced epiphyseal bone damage in *Fas −/−* mice with AIA due to preserved pool of early osteoprogenitor cells and sustained capability to form bone in inflammatory conditions (Lazić Mosler et al., 2019). We proposed that immune disturbances preceding bone loss are also affected by absence of functional Fas. Therefore, in the synovial compartment of wt and *Fas −/−* non-immunized (NI), immunized control (IMM) and arthritic (AIA) mice, we assessed the proportions of CD3^+^ T lymphocytes, considered responsible for the immune-mediated pathology, CD11b^+^Gr-1^+^ granulocytes-monocytes and F4/80^+^macrophages involved in the inflammatory component of arthritis (Figure 1A).

The proportions of all populations were higher AIA wt mice, in comparison to all other experimental groups (Figure 1B). The proportion of CD11b^+^Gr-1^+^ myeloid cells was significantly higher in the synovial compartment of IMM wt mice in comparison to NI group, with additional increase in wt AIA mice, which was absent in *Fas −/−* mice (Figure 1B), pointing to the fact that Fas deficiency suppresses accumulation of CD11b^+^Gr-1^+^cells in both conditions, general immunization and AIA. A similar trend was observed for F4/80^+^cells. Proportions of CD11b^+^Gr-1^+^ and F4/80^+^cells were positively associated with joint swelling, but only CD11b^+^Gr-1^+^ revealed a strong negative association to the epiphyseal bone volume (Table 1). While T lymphocytes were weakly positively associated with joint swelling (Figure 1B), their increase with immunization and arthritis was not affected by Fas deficiency (Table 1).

**TABLE 1:** Associations of proportions of synovial hematopoietic cell populations with knee diameter and bone volume in antigen-induced arthritis.

		CD3^+^ (%)	F4/80^+^ (%)	CD11b^+^Gr-1^+^ (%)
Knee diameter (mm)	ρ^*^ P N	0.434 **0.0029** 45	0.558 **0.0001** 45	0.647 **<0.0001** 45
BV/TV (%)	ρ P N	-0.277 0.0650 45	0.407 **0.0056** 45	-0.658 **<0.0001** 45

Proportions of hematopoietic populations are determined by flow cytometry and expressed as a proportion of single live cells; knee diameter was measured by caliper; BV/TV, percent bone volume, determined for distal femoral epiphyseal trabecular bone by µCT; *Spearman's correlation coefficient; statistically significant associations are marked in bold text..

### 3.2 Myeloid cells from *Fas −/−* mice differentially express several genes

**FIGURE 2:**
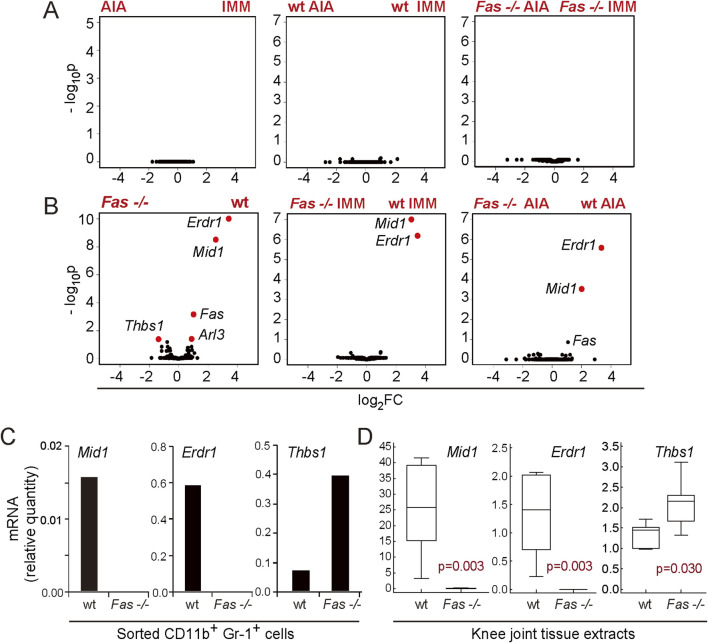
Differential gene expression analysis of synovial myeloid (CD11b^+^Gr1^+^) populations from immunized wild-type (wt) and Fas-deficient (*Fas −/−*) mice on day 10 of antigen-induced arthritis. Volcano plots showing negative logarithm of adjusted p values (-log_10_p), in relation to the logarithm of gene expression fold-change (log_2_FC) for differential gene expression analysis between mice with arthritis (AIA) and immunized control mice (IMM) **(A)** and *Fas −/−* mice and wt mice **(B)**. Markers of individual genes with significant adjusted p values (Benjamini-Hochberg, BH, p < 0.05) and fold change (absolute FC > 1.5) are marked red. **(C)** Validation of microarray data by RT-PCR analysis of *Erdr1*, *Mid1*, and *Thbs1* expression in pooled sorted CD11b^+^Gr-1^+^ cells from wt (n = 5), and *Fas −/−* mice (n = 4) with arthritis (AIA), and knee tissue extracts **(D)** of wt (n = 6) and *Fas −/−* AIA mice (n = 7, right panels). Samples for microarray were selected from three separate experiments, based on the RNA quality. Validation of microarray results was performed in two separate experiments, one for analysis of sorted cells and one for the analysis of tissue extracts. Mice were sacrificed on day 10 after arthritis induction. Gene expression is normalized to the *β-actin* expression. Horizontal line, median; boxes, IQR; whiskers, range; statistical significance is stated on plots (p < 0.05, Mann-Whitney test).

**FIGURE 3:**
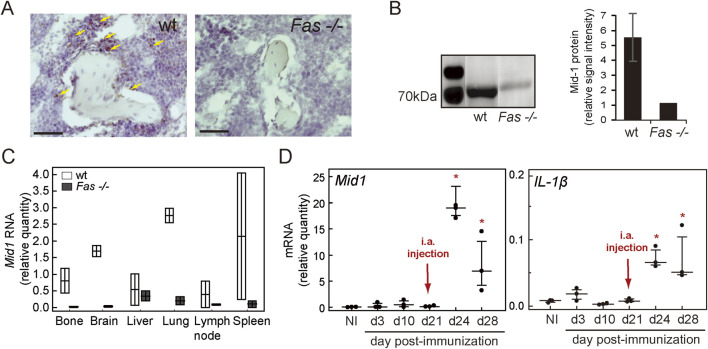
Expression of Mid-1 over the course of AIA. **(A)** Expression of *Mid1* RNA in the knee joints of wt and *Fas −/−* mice with arthritis, assessed by *in situ* hybridization (RNAScope). Arrows, brown signal resulting from binding of *Mid1* probe, bars, 50 µm. Slides for RNA Scope were selected from a representative experiment used for histology and µCT analysis. Three joints from wt and *Fas −/−* mice with arthritis were hybridized, with similar results, and representative images are shown. **(B)** Western blot analysis of Mid-1 expression in knee tissue extracts of wt and *Fas −/−* AIA mice, and analysis of Mid-1 signal intensity, normalized to total protein signal intensity, acquired from membranes using stain-free technology (Rivero-Gutiérrez et al., 2014). Protein extraction was performed from 6 wt and 2 Fas−/− knees harvested from one experiment. **(C)** Expression of *Mid1* in various tissues of wt (n = 2) and Fas −/− mice (n = 2) with AIA. Tissues were harvested from a single experiment. **(D)** Expression of *Mid1* and *IL-1β* in total joint tissue extracts of wt mice during immunization (d3-21), early (d24), and 1 week after arthritis induction (d28) in a single experiment. Gene expression was determined by RT-PCR and normalized to the expression of *β-actin*, n = 3, markers, individual values, horizontal lines, median (IQR), * denotes p < 0.05, in comparison to NI group (Kruskal-Wallis test).

As the differences in proportion of CD11b^+^Gr-1^+^ cells between wt and *Fas −/−* became evident only after immunization, we posited that comparing their transcriptomes in immunized wt and *Fas −/−* mice would reveal genes responsible for different response to immunization. Since we aimed to distinguish the myeloid cell-specific mediators of transition from immunized condition, characterized by accumulation of inflammatory innate cells, towards the onset of overt arthritis, which are altered by Fas deficiency, we analyzed the gene expression profile of CD11b^+^Gr-1^+^ cells from the synovial compartment of IMM and AIA wt and *Fas −/−* mice purified by FACS. Differential gene expression analysis revealed no differences in expression profiles of myeloid cells from IMM and AIA mice within wt and Fas −/− groups, or when comparing all samples regardless of genotype (Figure 2A), but we found an overexpression of midline 1 (*Mid1*) and erythroid differentiation regulator 1 (*Erdr1*), and an underexpression of thrombospondin 1 (*Thbs1*) when comparing wt mice to *Fas −/−* mice (Figure 2B). To validate the findings of microarrays, we analyzed the expression of those three genes by RT-PCR in sorted CD11b^+^Gr-1^+^ cells and knee joint tissue extracts from NI and AIA mice. In this group of experiments, we used NI mice to address potential changes in selected genes with immune activation. *Mid1* and *Erdr1* expression profiles were comparable in sorted CD11b^+^Gr-1^+^ cells (Figure 2C, upper panels) and knee joint extracts (Figure 2C, lower panels). Expression levels of *Erdr1* were similar in NI and AIA wt mice while *Mid1* was upregulated in wt AIA mice in comparison to the NI group, which pointed to its potential association with arthritis pathogenesis. *Thbs1*, similar to microarray data, had a lower expression in wt than in *Fas −/−* mice. The difference in expression of *Thbs1* was more pronounced in sorted CD11b^+^Gr-1^+^ cells, while strain-specific and disease-related differences were less pronounced in joint tissue extracts, pointing to other cellular sources of *Thbs1*. To further distinguish which of differentially expressed genes have a potential role in the pathogenesis of arthritis, we analyzed associations of candidates with knee joint diameters, and expression of proinflammatory cytokines IL-1β, tumor necrosis factor α (TNFα), and IL-6 in the knees of wt mice. Out of three validated genes, *Mid1* had the strongest association with joint swelling, and was also the only candidate whose expression was positively associated with expression of all three inflammatory cytokines (Supplementary Table S1). As assessed by *in situ* RNA hybridization of the sections of knee joints, expression of *Mid1* was abundant in subchondral juxta-trabecular bone marrow cells of wt AIA mice and absent in *Fas −/−* AIA mice (Figure 3A). Similarly to the gene expression level, the Mid-1 protein quantity was significantly reduced in *Fas −/−* mice in comparison to wt mice (Figure 3B). In addition, we performed a wider assessment of expression in various tissues of AIA mice and observed a generalized downregulation of *Mid1* in a multitude of tissues, except in the liver (Figure 3C). However, over the course of AIA, *Mid1* expression corresponded to the pattern of expression of proinflammatory IL-1β. During induction, both *Mid1* and *IL-1β* expression levels remain relatively low, with an increase in the early phase of arthritis, and persisting throughout the acute phase (Figure 3D).

### 3.3 Expression of Fas in CD11b^+^Gr-1^+^ myeloid cells is not required for proinflammatory activity of Fas in arthritis

**FIGURE 4:**
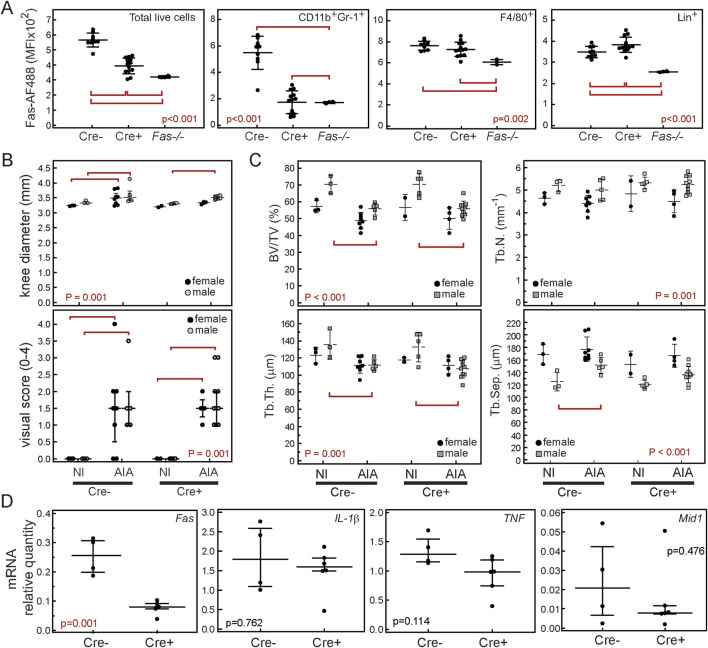
Myeloid-specific ablation of Fas does not ameliorate antigen-induced arthritis (AIA). Fas^fl/fl^LysMCre^+/−^ were crossed with Fas^fl/fl^ mice to produce littermates with conditional ablation of Fas in myeloid cells (Fas^fl/fl^LysMCre^+/−^, Cre^+^) and controls (Fas^fl/fl^LysMCre^−/−^, Cre^–^). Experiments were repeated 3 times and data are cumulative of minimum 2 representative experiments. **(A)** Bone marrow cells from Cre^−^, Cre^+^ and Fas −/− mice were stained with anti-Fas(CD95)-AF488 or corresponding isotype control, and CD11b-PE, Gr-1-PECy7, F4/80-APCCy7, and CD3/B220/NK1.1-APC antibodies. Mean fluorescence intensities (MFI) for each population are shown as individual values (markers), horizontal lines and bars are mean ± SD. Mice were sacrificed on a day 10 post-i.a. injection and arthritis was assessed by **(B)** measuring knee diameters and semi quantitative visual soring using appropriate scale (0-no arthritis, 1-discrete localized thickening of the joint capsule, 2-mild swelling, absence of sharp patellar ligament contour, 3-clear swelling with diffuse thickening of the joint capsule, 4-severe swelling and deformity, visible through the skin). **(C)** Subchondral epiphyseal bone volume was assessed by µCT in Cre-NI^−^ (n = 7 4m/3f), Cre^−^AIA (n = 13, 5m/8f), Cre^+^NI (n = 6, 4m/2f), Cre^+^AIA (n = 14, 10m/4f). The following variables were analyzed in distal femoral epiphyses: trabecular bone volume (BV/TV, %), trabecular number (Tb.N., mm^-1^), trabecular thickness (Tb.Th., µm), and trabecular separation (Tb.Sep., µm). **(D)** Expression of *Fas, TNF, IL-1β* and *Mid1* mRNA in total knee joint tissue from Cre^−^ mice with AIA (Cre^−^, n = 4, 2m/2f), and Cre^+^ mice with AIA (Cre^+^, n = 6, 4m/2f). Markers represent individual values, horizontal lines and error bars are mean ± SD (A, B top panel, C) or median (IQR) (B bottom panel, D); statistical significance is marked on plots with red lines connecting experimental groups with p < 0.05 (ANOVA and Student-Newman-Keuls *post hoc* test, A, B top panel, C; Kruskal-Wallis test, B bottom panel; Mann-Whitney test, D).

Since we described a decreased accumulation of myeloid cells as a result of global inactivation of *Fas*, we posited that this subset requires the expression of functional Fas to home and accumulate in joints during immunization and arthritis induction. Therefore, we hypothesized that myeloid-specific deletion of *Fas* using LysMCre mice would block their accumulation and abolish their expression of *Mid1*. We first assessed the efficacy of *Fas* deletion in various bone marrow subsets, by comparing the expression of Fas on CD11b^+^Gr-1^+^ myeloid cells, F4/80^+^ macrophages, and lymphoid lineage CD3^+^B220^+^NK1.1^+^ cells from mice with conditional ablation of *Fas* driven by the LysM promoter (Cre^+^) with cells from the control mice (Cre^−^) and cells from mice with a global Fas deficiency (*Fas −/−*). Expression of Fas in CD11b^+^Gr-1^+^ myeloid lineage from Cre^+^ cells was comparable to the levels observed in mice with a global Fas deficiency, but a residual expression was detected on the F4/80^+^ macrophage subset (Figure 4A). As expected, the lymphoid lineage was not affected by the conditional ablation driven by the LysM promoter (Figure 4A). Both, Cre^+^ and Cre^−^ mice showed signs of overt arthritis such as increased joint diameters and arthritis visual scores (Figure 4B), as well as a histological presence of a synovial infiltrate and cellular exudate (not shown). This was accompanied by decreased distal epiphyseal BV/TV and trabecular thickness (Figure 4C), revealing the lack of protective effect of *Fas* inactivation in CD11b^+^Gr-1^+^ cells. In addition, expressions of proinflammatory cytokines and *Mid1* were comparable in Cre^+^ and Cre^−^ mice with AIA confirming that expression of Fas in CD11b^+^Gr-1^+^ myeloid cells is not necessary for their infiltration and upregulation of *Mid1* expression (Figure 4D).

### 3.4 Soluble Fas agonist promotes expression of *Mid1* and proinflammatory cytokines

**FIGURE 5:**
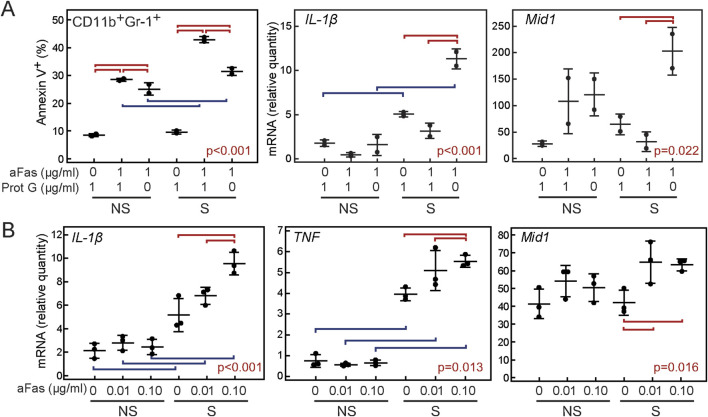
Proinflammatory and apoptotic effect of anti-Fas antibody on cells of myeloid (CD11b^+^Gr1^+^) lineage. Inflammation was induced by 2 μg/mL heat-inactivated *Mycobacterium tuberculosis*, and after incubation for 1 h/37°C, appropriate combinations of anti-Fas antibody, isotype control antibody, and protein G were added. After incubation for 18h/37°C cells were harvested for flow cytometry and/or RNA isolation. **(A)** Proportion of apoptotic (annexin V^+^) cells, and expression of proinflammatory *IL-1β* and *Mid1* in groups of non-stimulated (NS) and inflammatory-stimulated cells (S) treated with 1 μg/ml isotype control antibody (NI IgG) and/or 1 μg/ml protein G (Prot G) and/or 1 μg/ml anti-Fas antibody (aFas), **(B)** Expression of proinflammatory cytokines in groups of non-stimulated (NS) and inflammatory-stimulated cells (S) treated with low doses (0.01–0.1 μg/ml) of anti-Fas antibody. 10^6^ cells were plated per well of a 96-well culture plate. Cells were grown in duplicates (n = 2, A) or triplicates (n = 3, B), and experiments were repeated three times with similar results. Markers, individual values, horizontal lines, mean ± SD. Statistically significant difference within groups of S and NS cells is marked with red lines connecting experimental groups with p < 0.05, statistically significant difference between identically treated S and NS cells is marked with blue lines connecting experimental groups with p < 0.05, p values are marked on plots (ANOVA and Student-Newman-Keuls post-hoc test).

To assess the involvement of Mid-1 in Fas-dependent inflammatory activation, we analyzed *in vitro* responses of bone marrow cells from wt mice to different modes of Fas activation in resting non-stimulated cells and cells stimulated with heat-inactivated *M. tuberculosis* as a component of CFA, used to induce inflammatory activation in AIA. The first set of experiments sought to assess how various conditions of cell treatment with agonistic anti-Fas antibody affect their inflammatory response. Cells treated with the antibody and protein G exhibited an apoptotic response, observed by increased annexin V labeling in flow cytometry, which was most pronounced in CD11b^+^Gr-1^+^ cells. However, cells treated with agonistic anti-Fas antibody in absence of protein G displayed reduced apoptotic response accompanied by a dose-dependent increase in the expression of *IL-1β, TNF* and *Mid1* genes (Figures 5A,B).

### 3.5 Inactivation of Mid1 has a variable anti-inflammatory effect *in vitro*

**FIGURE 6:**
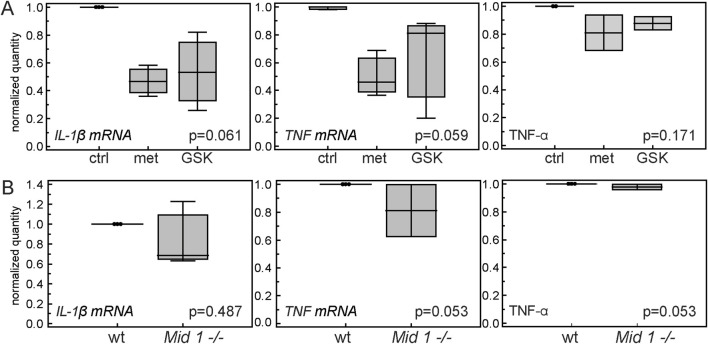
Effects of Mid-1 inactivation on the expression of pro-inflammatory cytokines in inflammatory-stimulated cells. **(A)** Pharmacological inhibition of Mid-1 in inflammatory-stimulated bone marrow cells. Cells were pre-treated with 20 µM metformin or 20 µM of Mid-1 peptide antagonist (GSK'364A) for 1h/37°C which was followed by the addition of heat inactivated *Mycobacterium tuberculosis* in final concentration of 10 µg/µL. After overnight incubation at 37°C, expression of proinflammatory cytokines *IL-1β*, and *TNF* and concentration of TNF*-α* were assessed by RT-PCR and ELISA. Experiments were performed in duplicates to quadruplicates, and values are calculated as ratios of means of expression/concentration of cytokine in the stimulated cells treated with metformin or GSK'364A over means of expression/concentration of cytokine in stimulated untreated cells in each experiment (n = 3). **(B)** Expression of inflammatory cytokines in inflammatory-stimulated bone marrow cells from wt and *Mid1 −/−* mice treated with agonistic anti-Fas antibody. Cells were treated with 10 µg/µL heat-inactivated *Mycobacterium tuberculosis* and 0.1 µg/mL anti-Fas antibody. After overnight incubation at 37°C, expression of proinflammatory cytokines *IL-1β*, and *TNF* and concentration of TNF*-α* were assessed by RT-PCR and ELISA. Experiments were performed in duplicates to quadruplicates, and values are calculated as ratios of means of expression/concentration of cytokine in the stimulated cells treated with metformin or GSK'364A over means of expression/concentration of cytokine in stimulated untreated cells in each experiment (n = 3). Horizontal line, median; boxes, IQR; whiskers, range; p values are marked on plots (A, Kruskal-Wallis test; B, Mann- Whitney test).

After we established that low doses of Fas agonist in the absence of protein G may act proinflammatory via stimulation of *Mid1* expression, we hypothesized that inactivation of Mid-1 would have an anti-inflammatory effect. We first assessed whether pharmacological inactivation of Mid-1 will reduce proinflammatory cytokine production in bone marrow cells stimulated with heat-inactivated *M. tuberculosis*. We hypothesized that proinflammatory effect of Mid-1 is mediated by the increased proteasomal degradation of anti-inflammatory PP2A through the interaction with its regulatory subunit α4 (Trockenbacher et al., 2001). This interaction can be blocked by metformin (Kickstein et al., 2010; Demir et al., 2014) or specific antagonistic peptide GSK′364A (Monteiro et al., 2018). Treatment of activated bone marrow cells with both metformin and GSK′364A variably inhibited transcription of proinflammatory *IL-1β* and *TNF*, and reduced the level of TNF-α protein, but this effect was not consistent among repeated experiments (Figure 6A). The more pronounced effect of metformin could be attributed to its multiple anti-inflammatory mechanisms (Kristófi and Eriksson, 2021). In the next set of experiments, we assessed whether genetic inactivation of *Mid1* would have the ability to block the proinflammatory cytokine expression in anti-Fas-stimulated bone marrow cells. Cells from *Mid1 −/−* mice revealed a variable decrease in expression of proinflammatory cytokines (Figure 6B), so we concluded that Mid1 signaling is not the exclusive mechanism underlying the proinflammatory effects of Fas.

### 3.6 Inactivation of *Mid1* partially mitigates AIA

**FIGURE 7:**
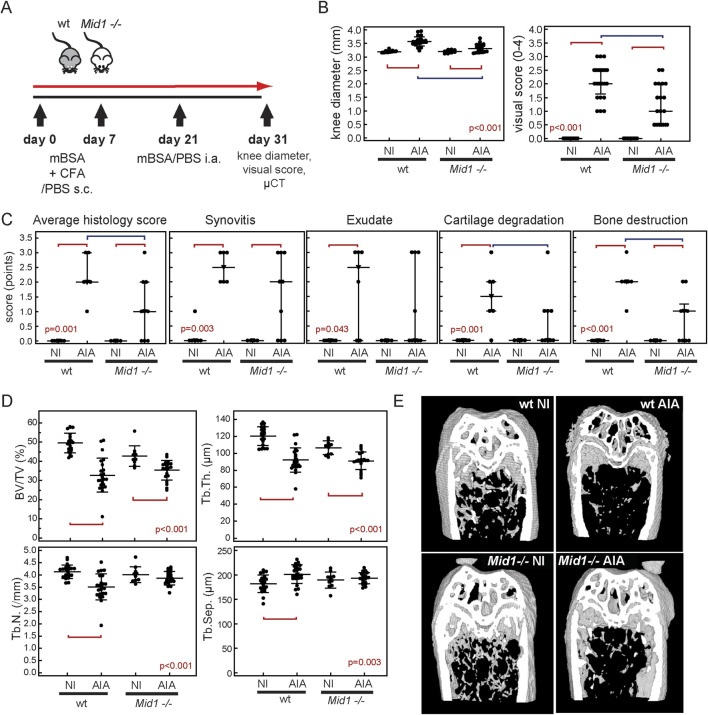
Mid-1 inactivation moderately alleviates antigen-induced arthritis (AIA). **(A)** AIA was induced in mice with a gene knockout for *Mid1* (*Mid1 −/−*) and wt mice. Visual score, knee diameters and µCT values represent cumulative data from 4 separate experiments, while histology score was assessed in one of the representative experiments. Mice were sacrificed on a day 10 post-i.a. injection and arthritis was assessed by **(B)** measuring knee diameters and semi quantitative visual soring in wt NI (n = 21), wt AIA (n = 23), *Mid1 −/−* NI (n = 10), *Mid1 −/−* AIA (n = 20) using appropriate scale (0-no arthritis, 1-discrete localized thickening of the joint capsule, 2-mild swelling, absence of sharp patellar ligament contour, 3-clear swelling with diffuse thickening of the joint capsule, 4-severe swelling and deformity, visible through the skin). **(C)** Total histology score in wt NI (n = 6), wt AIA (n = 6), *Mid1 −/−* NI (n = 4), and *Mid1 −/−* AIA (n = 9). Scoring was performed according to synovial thickening, presence of exudate in joint space, cartilage degradation, and subchondral bone damage, according to the 3 point scale. **(D)** Subchondral epiphyseal bone volume was assessed by µCT in wt NI (n = 21), wt AIA (n = 23), *Mid1 −/−* NI (n = 10), *Mid1 −/−* AIA (n = 20). The following variables were analyzed in distal femoral epiphyses: trabecular bone volume (BV/TV, %), trabecular number (Tb.N., mm^-1^), trabecular thickness (Tb.Th., µm), and trabecular separation (Tb.Sep., µm). **(E)** Representative 3D models of distal femora, from µCT reconstruction images. Markers represent individual values, horizontal lines and error bars are mean ± SD (B left panel, C) or median (IQR) (B right panel, D); statistical significance is marked on plots with red lines connecting experimental groups with p < 0.05 (ANOVA and Student-Newman-Keuls *post hoc* test, B left panel, C; Kruskal-Wallis test, B right panel, D).

We further assessed the effect of *Mid1* inactivation on arthritis in *Mid1 −/−* mice. *Mid1 −/−* mice developed AIA but joint swelling and arthritis visual score were reduced in comparison to wt AIA mice (Figures 7A,B). The overall histology score, synovial infiltration and bone destruction were clearly increased in *Mid1 −/−* mice in comparison to control NI *Mid1 −/−* mice, but the overall score, cartilage degradation and bone resorption were also significantly lower in comparison to wt AIA mice (Figure 7C). Distal epiphyseal trabecular bone volume and trabecular thickness were reduced in both wt and *Mid1 −/−* mice in comparison to NI controls (Figures 7D,E). However, a significant reduction in trabecular number and increase in trabecular separation were observed in wt mice with AIA, while the number and separation of distal epiphyseal trabeculae in *Mid1 −/−* mice with AIA were similar to corresponding controls. Taken together these results point to a partial protective effect of *Mid1* inactivation on cartilage and bone damage in inflammatory arthritis.

## 4 Discussion

This study demonstrated that expression of functional Fas is required for activation and recruitment of myeloid cells to the affected joints in the murine AIA. Wild-type mice showed increased accumulation of myeloid cells, particularly of CD11b^+^Gr-1^+^ cells even in immunized mice without arthritis, suggesting a role of Fas in their accumulation prior to the arthritis induction by antigen challenge. We also confirmed that the proinflammatory effect of low doses of Fas agonistic antibody is particularly evident on myeloid-lineage cells which points to potential non-apoptotic signaling during AIA (Audo et al., 2014; Buchbinder et al., 2018). Myeloid cell activation during immunization is an important factor for the induction of various experimental disease models based on immunization, such as collagen-induced arthritis and experimental autoimmune encephalomyelitis (Billiau and Matthys, 2011; Rumble et al., 2015; Morell et al., 2017), so functional Fas might be generally required for inflammatory cell activation. Transcriptomes of CD11b^+^Gr-1^+^cells in AIA and IMM mice were similar, suggesting their role in creating the inflammatory environment, required for successful induction of immune-mediated joint destruction. However, as the absence of CD11b^+^Gr-1^+^ accumulation is one of the main characteristics of attenuated AIA of Fas −/− mice, we proposed that potential molecular drivers of myeloid accumulation are intrinsically differentially expressed. Although the transcriptomes of wt and *Fas −/−* myeloid cells did not differ in a large number of genes, we identified three putative candidates and validated their expression pattern by RT-PCR. Among them, *Mid1* had the strongest association with inflammation markers and its expression was clearly upregulated with the onset of arthritis, suggesting its involvement in the pathogenesis of AIA. *Mid1* encodes a microtubule-related E3 ubiquitin-ligase, whose mutation in humans and mice results in developmental anomalies of CNS (Robin et al., 1996; Lancioni et al., 2010). The best described ubiquitination target of Mid1 is PP2A, responsible for dephosphorylation of mitogen-activated protein kinases (MAPK) (Trockenbacher et al., 2001). A deficiency of PP2A leads to increased phosphorylation of MAPK (p38, ERK, JNK) and NF-κB (IKKα/β, NF-κB p65) pathways in bone marrow macrophages, and stimulates the production of TNF-α, IL-6, and IL-10, which suggests its importance in limiting the inflammatory polarization of myeloid cells (Sun et al., 2017). Several studies have documented the involvement of Mid1 in immune regulation. Increased expression of *Mid1*, dependent on functional TLR4 and TNF-related apoptosis inducing ligand (TRAIL), has been reported in murine bronchial epithelium after exposure to house dust mite or rhinovirus infection. Silencing of *Mid1* or pharmacological activation of PP2A decreased the intensity of inflammation, accumulation of inflammatory cells, levels of IL-25, IL-33, CCL20, IL-5, IL-13, NF-κB activity and MAPK phosphorylation (Collison et al., 2013). The same research group reported similar results in the model of experimental allergic esophagitis, with disease amelioration in TRAIL −/− mice (Collison et al., 2015) and proposed a disease-promoting role of Mid-1 downstream of TRAIL in the pathogenesis of pulmonary fibrosis (Collison et al., 2019). TRAIL belongs to the same family as Fas ligand, and signals through TNF family death receptors (DR)5 and DR6, via similar signaling mechanisms as Fas, which suggest a downstream role of Mid-1 in death receptor signaling. Furthermore, Mid1 has been described as an important factor for functioning of cytotoxic T lymphocytes, by affecting their TCR signaling, exocytosis of lytic granules, polarization and migration (Boding et al., 2014a; Boding et al., 2014b). Recent study documented the role of Mid-1 in regulation of T cell entrance to the CNS in a model of experimental autoimmune encephalomyelitis and the ability of Mid-1 to affect mTOR signaling in T lymphocytes (Wei et al., 2024). In addition, apart from immune cells Mid-1 seems to signal through PP2A in endothelial cells, enhancing their expression of ICAM-1 and neutrophil adhesion and extravasation in septic conditions (Du et al., 2023).

We established that expression of Fas in CD11b^+^Gr-1^+^ subset is not necessary for their accumulation in affected joints and induction of AIA, pointing to predominance of proinflammatory over proapoptotic effects. Similar results were obtained by Huang and coworkers who studied the K/BxN model of murine arthritis and observed a comparable onset but faster resolution of arthritis in mice with a myeloid-specific deletion of Fas. This study reported a decreased frequency of CD11b^+^Gr-1^+^ cells in joints from Cre^+^ mice without apparent differences in migration or apoptosis of Fas-deficient CD11b^+^Gr-1^+^ cells. The faster resolution of arthritis was attributed to altered activation of Fas-deficient macrophages by IL -1β and the endogenous TLR2 ligand gp96 and overexpression of anti-inflammatory IL-10 (Huang et al., 2014). Although we assessed arthritis in subacute phase, we did not observe decreased inflammation or joint destruction. This could be also attributed to preserved expression of Fas in macrophages in our model, or differences between K/BxN and AIA models. In addition, even though myeloid cells represented 40%–50% of the total articular population and the conditional deletion of Fas was reflected in an overall decrease of Fas expression in total joint extracts, we did not observe decreased expression of *Mid1*. Thus, in the absence of generalized depletion of Fas, *Mid1* expression is not completely blocked. Among various tissues from Fas −/− mice, *Mid1* expression was reduced in lungs, lymph nodes, spleen, brain, bone and bone marrow with some residual transcriptional activity in liver. Lack of expression of *Mid1* and *Erdr1* could therefore be a result of constitutively inhibited transcriptional activity due to deficient Fas signaling or their inactivation by potential genomic alterations. Interestingly, *Mid1*, as well as *Erdr1* genes are located on the pseudoautosomal region (PAR) of sex chromosomes which represents the site of pairing and recombination between the X and Y chromosomes during male meiosis. It is genetically unstable, so deletion or duplication events involving various genes including *Mid1* and *Erdr1* are frequent in this region (Dal Zotto et al., 1998). Taking that into account poses the possibility that reduced Mid1 in Fas −/− mice might be a coincidental result of the PAR genetic instability rather than absence of the Fas signaling. Further studies are required to confirm this possibility.

Several studies described the ability of metformin to block the interaction of Mid1 and α4, a regulatory subunit of PP2A, and thus inhibit the proteasomal degradation of PP2A (Kickstein et al., 2010; Demir et al., 2014). A more specific inhibition of this interaction can be achieved by a peptide antagonist (GSK′364A) resembling the Mid-1-α4 binding site (Monteiro et al., 2018).

In our model, both metformin and GSK′364A had a variable inhibitory effect on TLR2-dependent transcription of *IL-1β* and *TNF* in primary murine bone marrow cells. Although proinflammatory activity of Mid-1 interaction with PP2A has been previously documented, inability of GSK′364A and metformin to completely abolish TLR2-dependent transcription of *IL-1β* and *TNF* suggests existence of additional, yet undiscovered downstream proinflammatory mediators of Mid-1. Recent studies reported Mid-1-dependent targeting of IRF3 and IFNAR2, acting as a negative feedback mechanism of the innate antiviral immunity (Chen X. et al., 2021; Chen et al., 2022). Moreover, deletion of *Mid1* decreased susceptibility of mice to viral myocarditis, pneumonia, and herpes simplex encephalitis by enhancing the production of type I IFN through its interaction with protein phosphatase 1A (PPM1A) responsible for dephosphorylation of TANK binding kinase 1 (TBK1). Phosphorylation of TBK1 is crucial for IRF3 transcription and production of IFN (Fang et al., 2022). In addition, one of the more recently defined Mid-1 ubiquitination targets is non-receptor tyrosine phosphatase PTP1B, whose degradation leads to overactivation of the STAT3 signaling pathway and promotes inflammation and fibrosis in diabetic kidney disease (Chen Q. et al., 2021).

Furthermore, in our model, genetic inactivation of *Mid1* did not completely abolish the cytokine production in inflammatory-activated bone marrow cells stimulated with anti-Fas agonistic antibody. This may implicate the existence of additional Mid-1-independent proinflammatory pathways or point to previously documented functional redundancy between Mid-1 and Mid-2 (Granata et al., 2005; Boding et al., 2015). Another potential explanation of inability of Mid-1 inhibition to completely abolish the described cytokine production is that we used total population of bone marrow cells to better model the complexity of interactions of multiple bone marrow-derived lineages, similarly to the interactions occurring during *in vivo* AIA, instead of purified myeloid populations. In this model, discrete effects within a specific lineage might be avoided or altered by individual variability in bone marrow composition.

In the AIA model, the inactivation of *Mid1* partially reduced bone and cartilage damage in AIA, without a significant effect on the synovitis and myeloid cell accumulation in affected joints. Our findings contrast the previous studies that reported proinflammatory activity of Mid-1 in lungs and other tissues (Collison et al., 2013; Collison et al., 2019; Wei et al., 2024; Du et al., 2023), and suggest that proinflammatory activity of Fas is independent of Mid-1. In addition, a recent study reported complete protection of *Mid1 −/−* mice from development of collagen-induced arthritis (Lin et al., 2024). According to this study Mid-1 promotes synoviocytes proliferation and migration by inducing ubiquitin-mediated proteasomal degradation of dipeptidyl peptidase-4. In our AIA model, overt synovitis was less frequent but not completely absent. The discrepancies between the results could be ascribed to differences in arthritis model, as well as to different approaches to induce *Mid1* deletion, so additional mechanistic studies are required to resolve these issues. The reports on direct effects of Mid-1 on bone structure and function are scarce. An increase in *Mid1* expression has been documented in autoimmune prone BXSB-Yaa mice, also characterized by alterations in bone structure (Namba et al., 2019). On the other hand, Mid-1 ubiquitinates RIC8 guanine nucleotide exchange factor A, which promotes the progression of osteoarthritis by activating p38 mitogen-activated protein kinase signaling pathway in chondrocytes, thus pointing to a protective effect of Mid-1 signaling in OA (Shen et al., 2021). All of the above points to multifaceted, not yet fully elucidated roles of Mid-1 in bone and cartilage homeostasis.

In conclusion, our results point to the requirement of functional Fas for the proinflammatory component of AIA, responsible for the recruitment and accumulation of innate inflammatory cells in arthritic joints. This accumulation is not driven by intrinsic factors expressed in the accumulated subset. Mid-1 aids in an inflammatory polarization of myeloid cells and promotes bone loss and cartilage degradation in arthritis.

## Data Availability

The original contributions presented in the study are publicly available. This data can be found here: Gene Expression Omnibus (GEO) repository, accession number GSE141592 at https://www.ncbi.nlm.nih.gov/geo/query/acc.cgi?acc=GSE141592.

## Abbreviations

AIA, antigen-induced arthritis; CFA, complete Freund’s adjuvant; IL, interleukin; IMM, immunized control mice; mBSA, methylated bovine serum albumin; MAPK, mitogen activated protein kinases; NF-κB, nuclear factor κB; NI, non-immunized control mice; PP2A, protein phosphatase 2A; TNFα, tumor necrosis factor α; TRAIL, tumor necrosis factor–related apoptosis inducing ligand; Mid-1, Midline 1 protein; Mid1, midline 1 gene.
