# Supplementary material for: Midline 1 associated with Fas signaling enhances murine antigen-induced arthritis
Source: Front Cell Dev Biol. 2025 May 15;13:1451093. doi: 10.3389/fcell.2025.1451093 (PMC12119625; doi:10.3389/fcell.2025.1451093)
Supplement: Supplementary file 1 [file DataSheet1.docx]

Supplementary Material

**Supplementary Table 1.** Associations of expression of midline 1 (*Mid1*), erythroid differentiation regulator 1 (*Erdr1*) and thrombospondin 1 (*Thbs1*) genes with knee diameter and expression of inflammatory cytokines in knee tissue extracts on a day 10 of antigen-induced arthritis.

|  | | Knee diameter  (mm) | IL-1^*^ | IL-6^*^ | TNF-α^*^ |
| --- | --- | --- | --- | --- | --- |
| Mid1^*^ | ρ^**^ P N | **0.675 0.0323** 10 | **0.782 0.0075** 10 | **0.697 0.0251** 10 | **0.709 0.0217** 10 |
| Erdr1^*^ | ρ P N | -0.298 0.4032 10 | -0.261 0.4671 10 | -0.200 0.5796 10 | -0.055 0.8810 10 |
| Thbs1^*^ | ρ P N | **-0.644 0.0443** 10 | -0.455 0.1869 10 | **-0.673 0.0330** 10 | -0.515 0.1276 10 |
| ^*^ relative mRNA quantity normalized to quantity of β actin  ^**^ Spearman's correlation coefficient | | | | | |


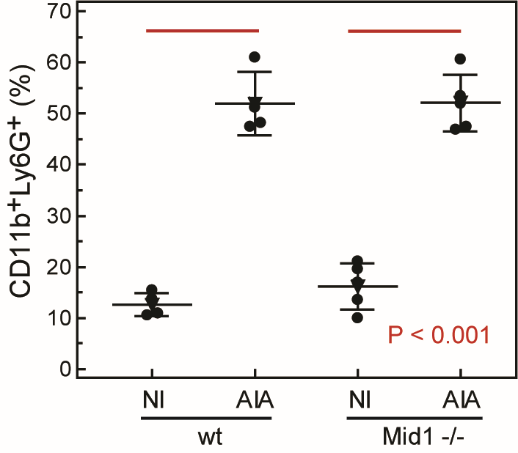
**Supplementary Figure 1. Myeloid populations in wild-type (wt) and Mid1-deficient mice (Mid1 –/–). (A)** Mice were sacrificed on day 10 following intra-articular (i.a.) injection, and single cell suspensions were labelled with anti-mouse Ly-6G-PE, anti-CD11b-PECy7, and 7-amino-actinomycin D (7-AAD). Proportions of granulocytes-monocytes (CD11b^+^, Ly-6G^+^), were determined amongst live cells. Horizontal lines and bars, mean±SD; p, one-way ANOVA
